# RB1 is the crucial target of the Merkel cell polyomavirus Large T antigen in Merkel cell carcinoma cells

**DOI:** 10.18632/oncotarget.8793

**Published:** 2016-04-18

**Authors:** Sonja Hesbacher, Lisa Pfitzer, Katharina Wiedorfer, Sabrina Angermeyer, Andreas Borst, Sebastian Haferkamp, Claus-Jürgen Scholz, Marion Wobser, David Schrama, Roland Houben

**Affiliations:** ^1^ Department of Dermatology, Venereology and Allergology, University Hospital Würzburg, Würzburg, Germany; ^2^ Department of Pharmacy, Center for Drug Research, University of Munich (Ludwigs-Maximilians-Universität), Munich, Germany; ^3^ Department of Dermatology, University of Regensburg, Regensburg, Germany; ^4^ Core Unit Systems Medicine, University of Würzburg, Würzburg, Germany

**Keywords:** Merkel cell carcinoma, polyomavirus, Large T antigen, retinoblastoma protein, viral carcinogenesis

## Abstract

The pocket protein (PP) family consists of the three members RB1, p107 and p130 all possessing tumor suppressive properties. Indeed, the PPs jointly control the G1/S transition mainly by inhibiting E2F transcription factors. Notably, several viral oncoproteins are capable of binding and inhibiting PPs. Merkel cell polyomavirus (MCPyV) is considered as etiological factor for Merkel cell carcinoma (MCC) with expression of the viral Large T antigen (LT) harboring an intact PP binding domain being required for proliferation of most MCC cells. Therefore, we analyzed the interaction of MCPyV-LT with the PPs. Co-IP experiments indicate that MCPyV-LT binds potently only to RB1. Moreover, MCPyV-LT knockdown-induced growth arrest in MCC cells can be rescued by knockdown of RB1, but not by p107 or p130 knockdown. Accordingly, cell cycle arrest and E2F target gene repression mediated by the single PPs can only in the case of RB1 be significantly reverted by MCPyV-LT expression. Moreover, data from an MCC patient indicate that loss of *RB1* rendered the MCPyV-positive MCC cells LT independent. Thus, our results suggest that RB1 is the dominant tumor suppressor PP in MCC, and that inactivation of RB1 by MCPyV-LT is largely sufficient for its growth supporting function in established MCPyV-positive MCC cells.

## INTRODUCTION

Several members of the polyomaviridae family (e.g. Simian Virus 40 (SV-40)) are capable of inducing tumor formation in animal models [1, 2], and the potential of SV40 to transform their host cells has been ascribed to the expression of viral oncoproteins, i.e. the T antigens (TA) [3]. Up to date, however, the Merkel Cell Polyomavirus (MCPyV) described in 2008 is the polyomavirus that is widely accepted to be causal for a human malignancy, namely Merkel Cell Carcinoma (MCC) [4, 5].

MCC is a highly aggressive skin cancer, and although it is relatively rare its incidence is increasing considerably [6]. Notably, in the vast majority of MCCs the MCPyV genome can be detected [7-9], and the observed clonal integration of the virus in the genome of the tumor cells [5] implies the causal relationship between MCPyV and MCC. This is further sustained by the addiction of MCPyV-positive MCC cells to expression of the T antigens [10], particularly due to a dependence on Large T antigen (LT) for MCC cell growth [11]. Interestingly, MCC-associated LTs are, due to stop codon mutations or pre-mature integration break points, generally truncated deleting the C-terminus required for viral replication but always preserving the LxCxE motif found in many proteins which interact with pocket proteins (PPs) [12, 13].

The PP family comprises three members, i.e. the Retinoblastoma protein 1 (RB1) and the two RB-like proteins p107 (RBL1) and p130 (RBL2). The family name refers to their binding ‘pockets’ mediating interaction with a multitude of other proteins [14]. All PPs bind to and thus regulate the activity of transcription factors of the E2F family. These interactions are regarded as central in controlling cell cycle progression from G1 to S phase [15]. Regulation of G1/S transition by PPs and E2Fs is a complex and at least partially redundant interplay of activator E2Fs (E2F-1, E2F-2, E2F-3a) preferentially binding RB1 and repressor E2Fs (E2F-3b, E2F-4, E2F-5) interacting with one or more of the PPs [16]. In normal quiescent cells, the PPs bound to E2Fs repress transcription of E2F-dependent promoters by different mechanisms; e.g. by recruiting histone deacetylases (HDACs) [17]. Upon phosphorylation by cyclin/cyclin-dependent kinase (CDK) complexes in late G1 PPs dissociate from their E2F partners, leading to transcription of S phase-specific genes [15].

Besides phosphorylation by CDKs, the suppressive function of the PPs can be halted by different viral proteins, such as HPV-E7, Ad-E1A and SV40-LT [18]. The binding of these oncoproteins via the conserved LxCxE motif results in disruption of repressive complexes of PPs with E2F family members leading to enhanced proliferation, and can thereby contribute to induction of cell transformation [18, 19].

Our previous finding, that the rescue of a TA knockdown-induced growth inhibition in MCC cells by ectopically expressed MCPyV-LT is dependent on an intact LxCxE motif suggested that PP inactivation is a critical function of MCPyV-LT in MCC [11]. Thus, here we address the questions which PPs are essential to be targeted by MCPyV-LT in MCC cells, and whether PP inactivation is sufficient for the growth promoting function of this viral protein in its natural tumor host cells. We provide evidence that inactivation of only RB1 by MCPyV-LT is essential and largely sufficient for supporting growth of MCC cells.

## RESULTS

### Homozygous deletion of the *RB1* gene in an MCPyV-positive cell line not depending on MCPyV-LT expression

**Figure 1:**
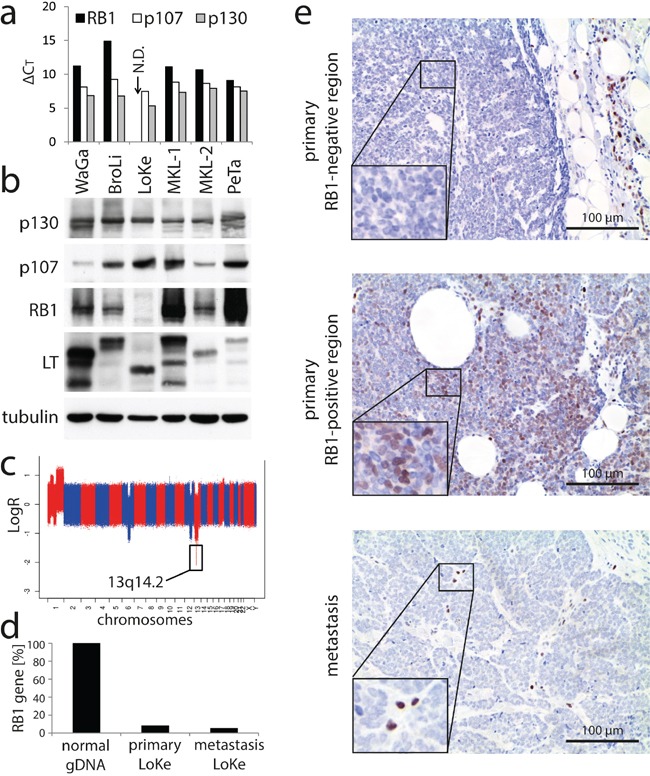
Loss of RB1 in the MCPyV-positive MCC cell line LoKe which is not depending on MCPyV-LT expression **a.** mRNA expression levels of the three PP family members were determined in the indicated cell lines by real-time PCR. ΔC_T_-values relative to the house keeping gene *RPLP0* (high values indicate low expression) are given. N.D.: not detectable. **b.** Immunoblot analysis of the PP protein expression levels in the indicated MCPyV-positive MCC cell lines. **c.** Microarray derived whole-genome copy number profile of the cell line LoKe, with x-axis coordinate representing positions along the genome. **d.** Relative quantification of the *RB1* gene by real time PCR in genomic DNA derived from the primary MCC tumor and in a subsequent metastasis of the respective patient excised 3 years later at the time when the LoKe cell line was derived from pleural effusion. Normal genomic DNA served as control. **e.** Immunohistochemical staining for RB1 in tissue sections of the two LoKe tumors described in d. Two different regions of the primary tumor are depicted.

In a first set of experiments we determined the expression of the pocket proteins in MCPyV-positive MCC cell lines. Real time quantitative PCR revealed that all PPs are expressed in almost all cell lines with generally higher mRNA levels for *p107* and *p130* than for *RB1* (Figure 1a). The only exception was the cell line LoKe for which no *RB1* expression could be detected. Notably, LoKe, although encoding a functional truncated MCPyV-LT [20], is up to date the only MCPyV-positive MCC cell line tested which is not dependent on LT expression for cell growth [21]. Immunoblot analysis confirmed the expression of all PPs in all other cell lines as well as the lack of RB1 expression in LoKe (Figure 1b).

Since real time PCR with genomic DNA suggested that lack of RB1 expression is due to a loss of the *RB1* gene (data not shown), we performed a comparative genomic hybridization for LoKe. This analysis revealed several genomic aberrations, with the relevant one being a very sharp homozygous deletion of the genomic region 13q14.2 (Figure 1c; basepairs 48.816.847 – 50.073.157 according to assembly GRCh37.p13) affecting only *RB1* and 10 additional genes (*CAB39L, CDADC1, CYSLTR2, FNDC3A, ITM2B, LPAR6, MLNR, PHF11, RCBTB2, SETDB2*).

The cell line LoKe was generated from a patient with metastatic MCC. Thus, to explore whether loss of RB1 had occurred after integration of MCPyV during tumor progression, we analyzed a metastasis excised at the time when the cell line LoKe was established and the primary tumor excised 3 years before. Real time PCR revealed largely reduced presence of the *RB1* gene in both tumors suggesting that at least the majority of tumor cells had lost both RB1 alleles. Immunohistochemistry on tissue sections revealed that in the metastasis all tumor cells were negative for RB1, in line with loss of both alleles of the *RB1* gene (Figure 1d). In contrast, in the primary tumor RB1 expression was heterogeneous with most parts lacking RB1 entirely (Figure 1d upper panel) while some minor areas demonstrated RB1 expression in a subset of tumor cells (Figure 1d middle panel). Sequencing of MCPyV-LT in genomic DNA derived from the primary tumor and several different metastases (including those analysed by immunohistochemistry) revealed that they all harboured the same unique stop codon present in the LoKe cell line (GenBank: KJ128381.1) implying that they are all clonally related.

### MCPyV-LT knockdown can largely be rescued by RB1 knockdown

**Figure 2:**
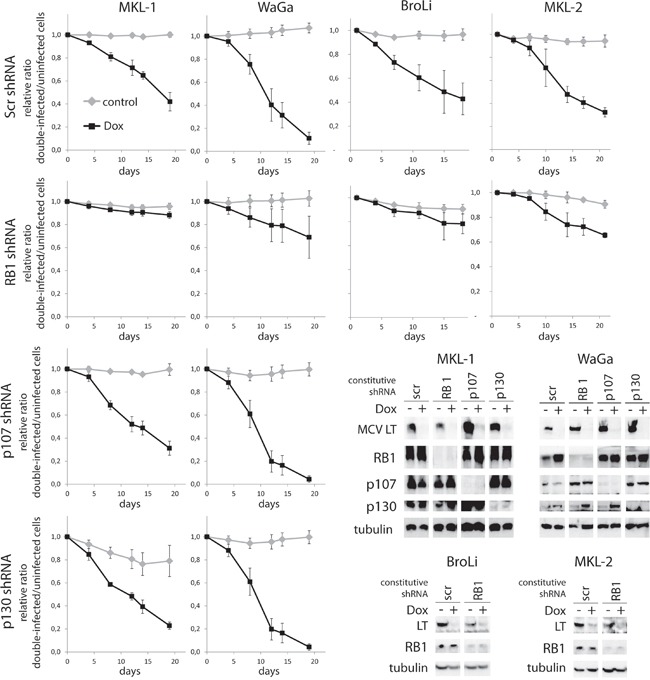
MCPyV-LT knockdown induced growth inhibition can be rescued by RB1 knockdown MCPyV positive cell lines MKL-1, WaGa, BroLi and MKL-2 transduced with a Dox-inducible TA.shRNA.tet vector system were infected with lentiviral shRNA constructs targeting RB1 (all cell lines), p107 or p130 (MKL-1 and WaGa). A Scr shRNA served as control. Pure populations infected with the PP shRNA constructs were established by antibiotic selection. Following 5 days of Dox treatment total cell lysates were harvested and analyzed by immunoblot for expression of MCPyV-LT and the different PPs. To evaluate changes in cellular growth, mixed populations of double-shRNA-infected cells characterized by green fluorescent expression and parental cells were cultured in presence or absence of Dox and changes of ratios were measured over time by flow cytometry. Mean values (+/− SD) of at least 3 independent experiments are depicted.

The LoKe cell line is characterized by loss of RB1 and independence of LT expression. In addition, analysis of the coding sequence of p107 and p130 demonstrated that both proteins are not affected by mutations (data not shown). These results suggest that inactivation of RB1 – but not the two other pocket proteins – is an essential function of MCPyV-LT in MCC cells. Consequently, to test whether RB1 inactivation might even be sufficient to substitute functionally for MCPyV-LT we performed shRNA knockdown experiments targeting MCPyV-LT and the different PP family members in MCC cells. To this end, we used the MCPyV-positive cell lines MKL-1, WaGa, BroLi and MKL-2 stably transduced with TA.shRNA.tet, a vector allowing Doxycycline (Dox)-inducible expression of an shRNA targeting all MCPyV-T antigen mRNAs [11]. We utilized the TA.shRNA instead of a LT-specific shRNA because the only effective LT-targeting shRNA exerts considerable off-target effects [11]. The TA.shRNA.tet cells were then stably transduced with a second shRNA vector constitutively expressing either a scrambled (Scr) or a shRNA targeting RB1. In addition, in the cell lines MKL-1 and WaGa shRNAs targeting p107 or p130 were applied in combination with the TA.shRNA to specifically analyze the role of all PP family members. Reduced expression of the PPs in response to the respective shRNA as well as Dox-induced knockdown of LT in these cell lines was monitored by immunoblot (Figure 2).

The impact of the shRNAs on growth properties of MCC cell lines was then analyzed by flow cytometry in mixed cultures of double-infected, green fluorescent und uninfected parental cells on the basis of GFP expression driven by the TA.shRNA.tet vector. Dox-induced LT-knockdown was associated with growth inhibition of cells expressing additionally the control Scr shRNA, indicated by a gradual loss of GFP-positive cells over time (Figure 2). In all four tested cell lines additional knockdown of RB1, however, resulted in a partial (WaGa, MKL-2) or even an almost complete rescue (MKL-1, BroLi) of impaired cell growth (Figure 2). For the interpretation of these data two of our previous observations are of importance. First, the TA shRNA induced growth arrest can be rescued to the same extent by an LT cDNA as by a TA gene (coding for sT and LT) indicating that with this experimental system we evaluate only LT functions although the applied TA shRNA also targets sT [20, 22]. Second, in the cell line WaGa the rescue by TA or LT is incomplete demonstrating a similar rescue activity as achieved by RB1 knockdown (Figure 2; [20, 22]). Hence, it is likely that the TA shRNA exerts growth inhibiting off-target effects in WaGa. Interestingly, RB1 and p130 are induced upon TA shRNA induction (Figure 2) potentially contributing to the incomplete rescue. In summary, RB1 loss is almost sufficient to substitute for MCPyV-LT in the cell lines WaGa, MKL-1 and BroLi and at least partially capable to rescue the loss of LT in MKL-2 cells. In contrast, knockdown of p107 or p130 did not affect the growth inhibition induced by LT knockdown in WaGa and MKL-1 cells (Figure 2).

**Figure 3:**
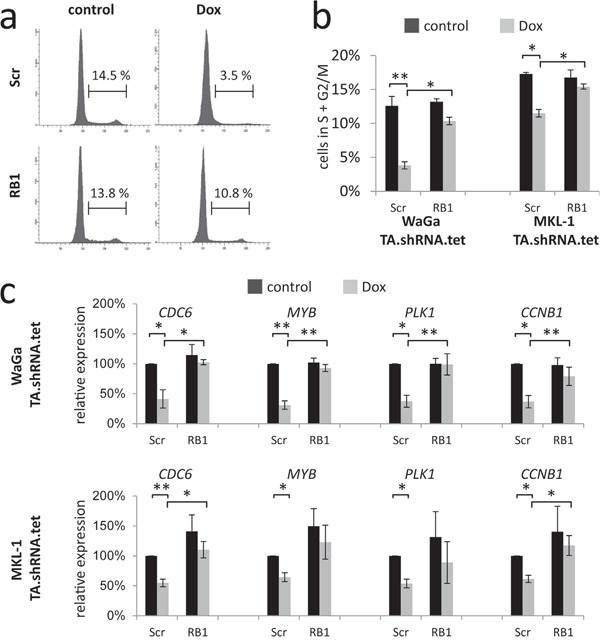
RB1 knockdown reverses TA knockdown-induced cell cycle arrest and E2F target gene repression WaGa and MKL-1 cells double infected with inducible TA-shRNA and constitutive Scr- or RB1-shRNA expression constructs were cultured for 5 days in the absence or presence of Dox. **a** and **b.** Fixed cells were stained with propidium iodide and DNA content was determined by flow cytometry. a) Examples of cell cycle profiles for WaGa. b) Depiction of the percentage of cells with >2N DNA. Bars represent mean values (+/− SD) of at least 3 independent experiments. **c.** Relative expression levels of the indicated cell cycle-related RB target genes were determined by real-time PCR and the ΔΔC_T_ method. *RPLP0* served as endogenous control for normalization and Scr shRNA-infected cells without Dox treatment were used as calibrator. Mean values (+/− SD) of 3 independent experiments are depicted. Statistical analyses were performed using paired student's t-test. (**p<0.005; *p<0.05).

To further evaluate these findings, and to exclude the possibility that paracrine effects distort proliferation measurements in the mixed culture assay [23], cell cycle analyses were performed in MKL-1 and WaGa cells following TA and RB1 knockdown. In accordance with the results of the mixed culture assay, TA shRNA-induced reduction of cells in S and G2/M phase could significantly be reversed by additional knockdown of RB1 (Figure 3a and 3b). Moreover, quantitative RT-PCR experiments revealed that TA shRNA-induced cell cycle arrest was associated with reduced expression of cell cycle related RB1 target genes *CCNB1, MYB*, *PLK1* and *CDC6* while upon additional knockdown of RB1 expression levels were hardly affected (Figure 3c).

### Strict overlap of genes regulated by MCPyV-LT and RB1 in MCC cells

**Figure 4:**
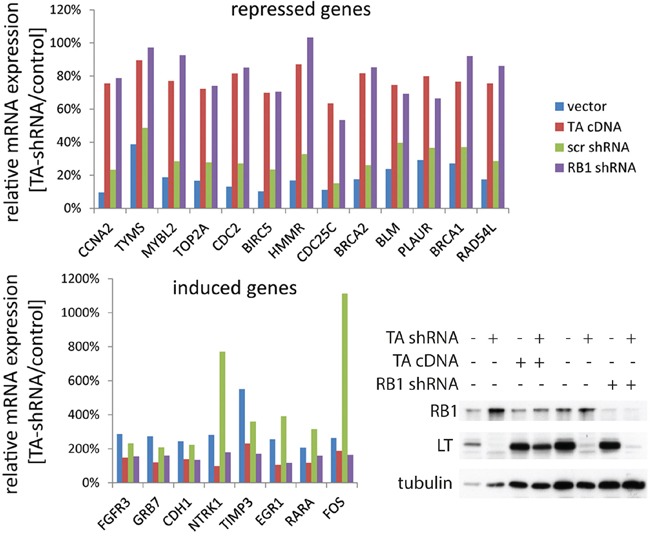
Overlap of genes regulated by MCPyV-TA and RB1 in MCC cells TA-shRNA was expressed in WaGa cells stably transduced with either empty vector, vector coding for MCPyV-TA, a Scr-shRNA vector or an RB1-shRNA construct. After 5 days LT and RB1 protein levels were analyzed by immunoblot, and mRNA expression levels of 245 cancer related genes were analyzed using the NanoString nCounter™ gene expression system [24]. 90 genes were excluded from further analysis due to very low expression. The absolute expression values of the remaining 155 genes were normalized to the mean value of the 6 house keeping genes. Depicted are the relative mRNA expression levels, i.e. TA-shRNA expressing cells relative to their controls, of the 21 genes displaying a more than two fold change in the empty vector and the Scr-shRNA cells.

To further scrutinize the extent RB1 inactivation can compensate for TA knockdown with respect to gene expression in MCC cells, we performed NanoString nCounter™ gene expression analyses [24]. To this end, the expression of 245 cancer related genes (+ 6 endogenous controls) was determined for mRNA derived from WaGa cells upon TA.shRNA expression. These cells were additionally stably transduced with either a construct coding for an shRNA-insensitive TA gene [11], with the RB1 shRNA or with the respective control vectors (cDNA vector; Scr shRNA). 90 genes demonstrating very low expression (less than 25 copies) were excluded from the analysis of differential expression upon TA.shRNA expression since for very rare mRNAs variability due to technical issues can be expected to be rather high [24]. From the remaining 155 cancer genes 21 gene demonstrated a more than 2-fold alteration in expression upon induced TA knock down, either downregulation (13 genes) or upregulation (8 genes), respectively (Figure 4). For all these 21 genes the TA.shRNA-induced changes were reversed by either TA re-expression or shRNA-mediated RB1 inactivation (Figure 4). Most of the genes downregulated following TA knockdown (e.g. *BIRC5 (survivin), BLM, CDC25a, BRCA 1 and 2, MYBL2, CCNA2, RAD54L, HHMR, TYMS*) have previously been described as E2F and/or RB1 target genes [25-28], a notion sustained by the observed re-expression upon RB1 knockdown (Figure 4). Others however, and in particular some of the genes upregulated upon TA.shRNA application (e.g. *PLAUR, FGFR3, TIMP3*) do not belong to the well established RB1 target genes. Nevertheless, re-expression upon RB1 knockdown suggests that these genes are at least indirectly regulated by RB1 in WaGa. Importantly, the observation that expression of every gene differentially expressed upon TA knockdown could be reversed by RB1 knockdown further supports that RB1 inactivation is the predominant function of truncated MCPyV-LT in MCC cells.

### MCPyV-LT preferentially interacts with RB1

**Figure 5:**
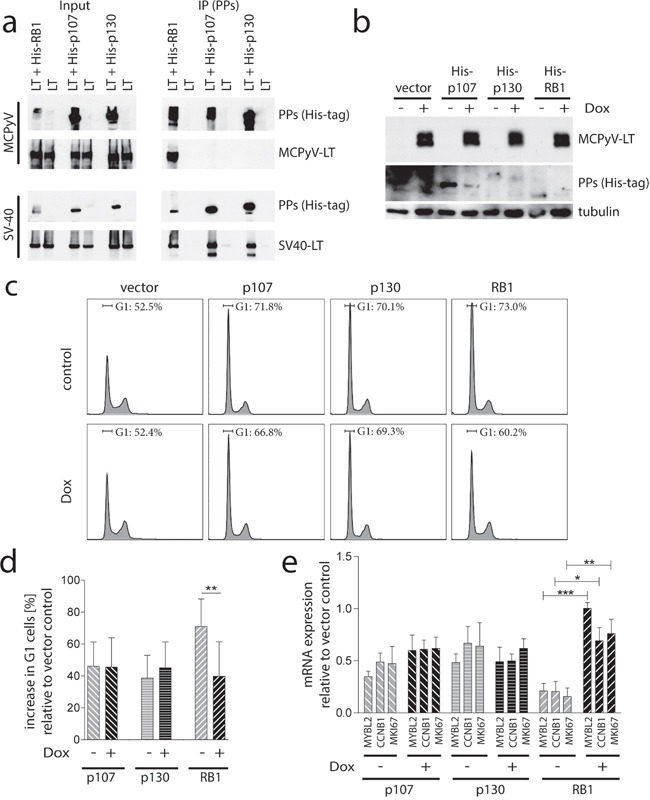
Preferential binding and inactivation of RB1 by MCPyV-LT **a.** Co-immunoprecipitation. His-tagged PPs were co-expressed with V5-tagged MCPyV-LT^278^ or SV40-LT in 293T cells. After 24 hours RB1 was immunoprecipitated with a His-tag-antibody. Co-immunoprecipitation of the LT proteins was analyzed by immunoblot using a V5-antibody. **b, c** and **d.** PP triple knockout mouse embryonic fibroblasts (MEF-TKO) [29] were manipulated for Dox-inducible expression of V5-tagged MCPyV-LT^278^. His-tagged versions of the different PPs were lentivirally transferred and 14 h later Dox-treatment was started. 24 hours later cells were fixed or lysed. b) Total cell lysates were subjected to immunoblot analysis using an α-His-antibody for detection of the PPs and an α-V5-antibody for the LT proteins c and d) Fixed cells were stained with propidium iodide and the percentage of cells in G1 was determined by flow cytometry. c) Representative cell cycle profiles are depicted. d) Mean values (±SD; n=6) of the increase in G1 cells relative to the vector control are displayed. **e.** Relative expression levels of the indicated E2F target genes were determined by real-time PCR. *muRPL37* served as endogenous control. Mean values (±SD; n=3) of the expression level relative to the vector control cells are depicted. Statistical analyses were performed using paired student's t-test. (***p<0.0005; **p<0.005; *p<0.05).

Our finding that RB1 inactivation is sufficient to rescue MCPyV-TA knockdown induced growth inhibition of MCPyV-positive MCC cells is surprising for two reasons. First, redundant functions of the PPs have been shown in many aspects, (e.g. unrestricted growth of fibroblasts can only be achieved by inactivation of all three pocket proteins [29]) and second, the related SV40-LT has been demonstrated to be capable of binding and inhibiting all three pocket proteins [30, 31]. Since the binding capacity of MCPyV-LT to RB1 is established [12, 32], we wondered whether MCPyV-LT can also bind to p107 and p130. Hence, transient co-expression of His-tagged versions of the three pocket proteins and V5-tagged SV40-LT or MCPyV-LT^278^ in 293T cells was followed by immunoprecipitations with an anti-His-tag antibody. As expected, SV40-LT co-immunoprecipitated with all three pocket proteins. In contrast, MCPyV-LT^278^ demonstrated a selective binding to RB1 (Figure 5a).

### MCPyV-LT fails to inhibit functionality of p107 and p130

A lack of co-immunoprecipitation cannot formally proof the absence of interaction between two proteins. We thus analyzed next whether MCPyV-LT is able to functionally interfere with the PPs. To address this question in a model system that allows unequivocal distinction of the different PPs, we utilized mouse embryonic fibroblasts derived from animals in which all three PP genes have been knocked out (MEF-TKO) [29] engineered for Dox-inducible expression of MCPyV-LT^278^ (Figure 5b). Ectopic expression of p107, p130 or RB1 in these cells (Figure 5b and Supplementary Figure S1) led to a partial cell cycle arrest (Figure 5c and 5d) as well as to reduced expression of the E2F target genes *MYBL2*, *CCNB1* and *MKI67* (Figure 5e). Upon induction of MCPyV-LT RB1-induced cell cycle arrest and gene repression were significantly reversed while MCPyV-LT did not affect the p107- and p130-induced effects (Figure 5c and 5d).

## DISCUSSION

The causal relation between MCPyV and MCC is widely accepted [33]. In this regard, although one study suggested that MCPyV is present in all MCCs [9], several other investigations imply that the entity MCC – as diagnosed by classical criteria – can be stratified into MCPyV-positive and MCPyV-negative cases [34-38]. Due to discrepancies in some of these reports it is not yet clear if the presence of the viral genome impacts clinical outcome of the disease. However, concerning molecular differences, several recent sequencing studies consistently reported that MCPyV-negative MCCs in contrast to MCPyV-positive cases are characterized by frequent deletions/mutations of the *RB1* gene [39-41]. Thus, RB1 inactivation seems to be an essential step in MCC development with inactivation occurring either genetically or in MCPyV-positive tumors by expression of a truncated LT with a generally preserved RB binding site [12, 13]. In the related SV40-LT this binding site contributes to LT's capability to inactivate all three pocket protein members, i.e. RB1, p130 and p107 [42].

MCPyV-LT, however, seems to discriminate between the different PPs. This is supported by our co-IP experiments in 293T cells as well as our functional data derived from co-expression of PPs and MCPyV-LT in triple PP knockout cells. We observed preferential binding of MCPyV-LT to RB1 compared to the two other PP family members, and demonstrated also a differential capability to interfere functionally with the different PPs. Indeed, co-expression of truncated MCPyV-LT can reverse cell cycle arrest and E2F target gene expression induced by RB1, but not the effects induced by p107 or p130 in MEF-TKO cells. This restricted interaction capacity with PPs distinguishes MCPyV-LT from LTs encoded by other polyomaviruses. Indeed, SV40-LT has the potential to abrogate RB1- as well as p130- and p107-induced gene repression and cell cycle arrest in RB1^−/ −^ Saos cells [43]. Moreover, also LT proteins from the human JC and BK polyomaviruses have been demonstrated to bind to all three PPs [44, 45]. Interestingly JC-LT exhibits the highest affinity for p107 while RB1 binding is relatively weak [45].

The observed preferential binding of RB1 by MCPyV-LT further expands the list of described differences such as that i) LT from MCPyV does not possess transforming capacity in fibroblast assays in contrast to e.g. SV40 (Shuda *et al.*, 2011) and ii) MCPyV-LT lacks a CKII consensus sequence at an important phosphorylation site which is present in SV40 and all human polyomaviruses harbouring an RB1 binding site (Schrama *et al.*, 2015). Differences between LT from MCPyV and those of SV40, JC and BK might reflect their assignment to different phylogenetic clades with MCPyV grouping with polyomaviruses found in chimpanzee, gorilla and bats [46].

In contrast to the many cellular interaction partners described for the well-studied SV40-LT [42], the number of proteins identified to interact with MCPyV-LT, i.e. RB1, HSC-70, Brd4 and Vam6p, is limited [12, 47-49]. Importantly, our findings in the current study suggest that in the natural tumor host cells MCPyV inactivation of RB1 appears to be the predominant and in some MCC cell lines the only essential function of MCPyV-LT to support growth of these cells. Indeed, knockdown of RB1 led in the tested MCPyV-positive MCC cell lines to a rescue of LT-knockdown-induced E2F target gene repression and more importantly, to a reversion of LT-knockdown-induced cell growth inhibition. The importance of the RB1-LT interaction is further sustained by a recent report: revealing that the overwhelming majority of MCPyV-LT induced gene expression alterations require the intact LxCxE binding motif [50].

Our findings on cell culture level pointing to a restricted but important interaction of MCPyV-LT with RB1 is supported by clinical findings. For example, LoKe is up to now the only studied MCPyV-positive MCC cell line not depending on expression of the viral LT protein despite the presence of an MCC-typical LT mutation preserving the RB binding motif. This suggests that LT and the RB-binding domain were required at some point during carcinogenesis [21]. The observed homozygous loss of the *RB1* gene in LoKe cells seems to render them independent of MCPyV-LT expression. The expression of wild type p107 and p130 in LoKe thus implies that inactivation of RB1 – but not the two other PPs – is an essential function of MCPyV-LT in MCC cells. Assuming equivalent molecular mechanisms in all MCCs, this is in line with the fact that inactivation of p107 and p130 in MCPyV-negative MCCs has not been reported [39, 40, 51]. Indeed, neither homozygous deletion/mutation of p107 and p130 nor mutation/copy number variations of upstream factors like p16^INK4A^, CDK4 or Cyclin D – which are common features of many tumor types [52] – have been described.

Regarding the molecular history of the MCPyV-LT-independent MCPyV-positive cell line LoKe co-presence of RB1 and MCPyV-LT in a portion of the neoplastic cells of the respective primary MCC tumor suggests that integration of MCPyV into the genome of the tumor cells preceded homozygous loss of RB1.

Although being essential for growth of established RB1 expressing MCC cells the role of MCPyV-LT in malignant transformation has not been finally established. In contrast to SV40-LT, MCPyV-LT is not transforming in fibroblast assays. Indeed, transforming capacity has been demonstrated only for MCPyV-sT so far, and could not be enhanced by MCPyV-LT [53]. In accordance, MCPyV-TA cannot induce a fully malignant phenotype in mouse models [54]. Fibroblast transformation *in vitro*, as well as induction of hyperproliferative lesions in mouse models by MCPyV-sT has been demonstrated to be dependent on a region called the LT stabilization domain, which is mediating the inhibition of different E3 ligases. Inactivation of protein phosphatase A the major function of SV40-sT seems not to be relevant [53, 55, 56]. Therefore, our observation that MCPyV-LT in contrast to SV40-LT - besides not binding p53 directly [32, 57] - discriminates between the different pocket proteins adds only one more piece to the puzzle of distinct features of these two oncogenic polyomaviruses.

These differences between SV40-LT and MCPyV-LT certainly contribute to their transforming capacity. In this regard, in some cellular systems inactivation of all three PPs is required to allow unrestricted growth [29]. Accordingly, many tumors require a broad disruption of the PP/E2F pathway by e.g. activation of cyclin-dependent kinases or inactivation of cyclin dependent kinase inhibitors [14]. MCC, however, seems to belong to a group of tumors, like small cell lung cancer and retinoblastoma in which inactivation of only RB1 is sufficient to allow tumor formation [52, 58]. The limited ability of MCPyV-LT to interfere with p107 and p130 may, therefore, account for a limited subset of cell types being transformable by MCPyV. Besides other factors (e.g. virus tropism) this may contribute to the fact that only the rare MCC and some subsets of chronic lymphocytic leukemia [59, 60] have been reported to be associated with this omnipresent virus. Finally, our data suggest that inactivation of RB1 is the only crucial function of MCPyV-LT to support growth of MCC cells.

## MATERIALS AND METHODS

### Ethics statement

This study was conducted according to the principles of the Declaration of Helsinki and analysis of patient derived samples was approved by the Institutional Review Board of Würzburg University Hospital (Ethikkommission der Medizinischen Fakultät der Universität Würzburg; sequential study number 124/05).

### Cell culture

The cell lines analyzed in this study include the MCPyV-positive MCC cell lines LoKe [21], PeTa [61], WaGa, BroLi, MKL-2 (all described in [10]) and MKL-1 [62] as well as the triple PP knock out mouse embryonic fibroblasts (MEF-TKO) [29]. HEK-293T cells were used for lentivirus production and for co-immunoprecipitation assays. All cell lines were grown in RPMI 1640 supplemented with 10% FCS, 100 U/ml penicillin and 0.1 mg/ml streptomycin. MEF-TKO cell were directly obtained from the lab were the cells were generated and characterized [29] and were not passaged for more than 6 months. All MCC cell lines as well as the HEK-293T cells were authenticated by STR profiling. Moreover, the MCC cell lines are routinely checked by sequencing for the presence of the characteristic Large T truncating mutations, which lead to a distinct molecular weight of the protein detectable by immune blotting (Figure 1).

### Vectors

For inducible knockdown of MCPyV-LT, we used the lentiviral single vector TA.shRNA.tet allowing constitutive GFP expression and Doxycycline (Dox)-inducible expression of an shRNA targeting all transcripts derived from the MCPyV early region [11]. For constitutive knockdown shRNA sequences targeting RB1, p107 or p130 (see Supplementary Table S1) were cloned into the lentiviral vector pGreenPuro. For Dox-inducible LT expression we used the two vector system Lenti-X Tet-On-3G (Clontech) with the cloning vector pLVX-Tre3G-IRES allowing inducible expression of two cDNAs from an internal ribosomal entry site (IRES)-containing transcript. Truncated MCPyV-LT^278^ was cloned into the cloning site preceding the IRES and GFP was inserted downstream of the IRES.

### Lentiviral infection

Lentiviral supernatants were produced in HEK293T cells using three (pRSV rev, pHCMV-G and pMDLg/pRRE) helper plasmids. Harvested virus supernatant was sterile filtered (0.45 μm) and polybrene was added (1 μg/ml) for infection. After 14-20 h incubation target cells were washed twice with medium and subjected to antibiotic selection.

### Mixed cell culture assay

Constitutive GFP expression from the TA.shRNA.tet construct was used to compare the growth behavior of double-infected and uninfected cells: TA.shRNA.tet cells were mixed with approximately 20% of untransduced cells, and changes in the frequency of GFP-positive TA.shRNA cells were recorded by flow cytometry over time.

### Cell cycle analysis

Cell were fixated in ice cold EtOH, treated with propidium iodide mix (PBS+ 1% FCS + 0.1 mg/ml propidium iodide + 0.1 mg/ml RNAse A) at 37°C for 1 h and analyzed by flow cytometry.

### Real time PCR

Total RNA was isolated with peqGOLD Total RNA Kit (PeqLab) and reverse transcribed using the Superscript II RT First Strand Kit (Invitrogen). Real time PCR was conducted in the ABI 7500 Fast Real-Time PCR cycler (Applied Biosystems) using a SYBR Green I Low Rox Mastermix (Eurogentec GmbH) and the respective primers (Supplementary Table S2). Following a 10 min denaturing step at 95°C 40 cycles with 15 seconds 95°C and 1 min 60°C were applied. Primer sequences and PCR efficiencies are given in Supplementary Table S2.

### Comparative genomic hybridization

DNA from MCC cell lines was hybridized to Affymetrix SNP 6.0 arrays, and data analysis was carried out with the Bioconductor package “copynumber”. Microarray data has been deposited at Gene Expression Omnibus (GSE73879).

### Immunohistochemistry

Three-micrometer sections of formalin fixed and paraffin embedded tumor tissues were stained as previously described [11] with an antibody targeting RB1 (G3-245; BD Pharmingen).

### NanoString nCounter™ gene expression analysis

100 ng total RNA were subjected to hybridization with the Nanostring Cref Kit (Cancer-Kit) containing probes for 245 cancer related gene products and the mRNAs of 6 house keeping genes. Following nCounter digital reading the values were normalized to the mean value of the house keeping genes.

### Immunoblotting

Immunoblotting was performed as previously described [11] The primary antibodies used in this study were directed against RB1 (G3-245; BD Pharmingen), p107 (sc-318; Santa Cruz), p130 (sc-317; Santa Cruz) MCPyV-LT (CM2B4; Santa Cruz), the V5 tag (SV5-Pk1; Abcam), His-tag (D3I1O; Cell Signaling) or β-tubulin (TUB 2.1; Sigma-Aldrich).

### Co-immunoprecipitation

293T cells were co-transfected with expression constructs coding for 6xHis tagged PPs and V5-tagged MCPyV-LT^278^ or V5-tagged SV40-LT. 24 hours after transfection cell lysates were harvested and Co-Immunoprecipitation was performed as recently described [63].

### Statistics

Student t test was performed with GraphPad Prism 5.03 software.

## SUPPLEMENTARY FIGURES AND TABLES


