# Supplementary material for: RB1 is the crucial target of the Merkel cell polyomavirus Large T antigen in Merkel cell carcinoma cells
Source: Oncotarget. 2016 Apr 18;7(22):32956–68. doi: 10.18632/oncotarget.8793 (PMC5078066; doi:10.18632/oncotarget.8793)
Supplement: Supplementary file 1 [file oncotarget-07-32956-s001.pdf]

RB1 is the crucial target of the Merkel cell polyomavirus Large T antigen in Merkel cell carcinoma cells

SUPPLEMENTARY FIGURE AND TABLES

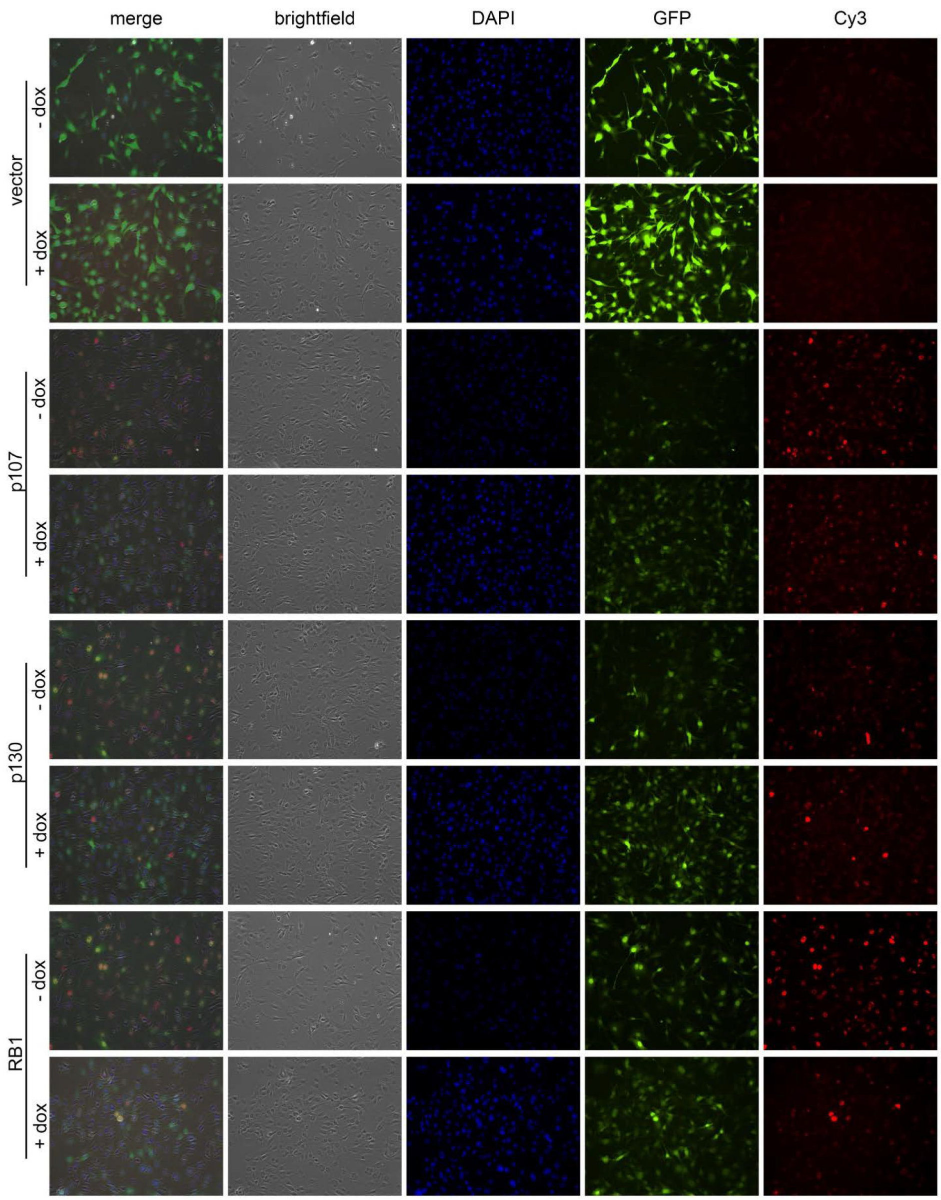

Supplementary Figure S1: (corresponding to Figure 5 b, c and d): Detection of ectopic PP expression in MEF-TKO cells by Immunofluorescence. PP triple knockout mouse embryonic fibroblasts (MEF-TKO) were manipulated for Dox-inducible expression of V5-tagged MCPyV-LT[278]. His-tagged versions of the different PPs in a vector co-expressing GFP were lentivirally transferred and 14 h later Dox-treatment was started. Following further 24 hours later cells were fixed with 4% formalin and subjected to immunofluorescence analysis using a His-Tag primary antibody and a Cy3 conjugated secondary antibody. Cell nuclei were stained with Dapi and by visualization of green fluorescence infection of the cells was monitored.

Supplementary Table S1: shRNA sequences

**MCPyV-TA**	ATCCACAAGCTCAGAAGTGACTTCTCTATTCAAGAGATAGAGAAGTCACTTCTGAGCTTGTGGAT
**RB1**	GTTAGGACTGTTATGAACACCTTGTTATGAACACCTTCCTGTCAGAGTGTTCATAACAGTCCTAAC
**p107**	GATGTTGTCAGTGAAAGAGCTCGAGCTCTTTCACTGACAACATC
**p130**	GAGCAGAGCTTAATCGAATTTCGAAAAATTCGATTAAGCTCTGCTC

Supplementary Table S2: Real time PCR primers

human			
	**forward**	**reverse**	**PCR efficiency**
**CCNB1**	CGCCTGAGCCTATTTTGGT	GCACATCCAGATGTTTCCATT	100,0%
**huCDC6**	CCTGTTCTCCTCGTGTAAAAGC	GTGTTGCATAGGTTGTCATCG	95,2%
**PLK1**	AAGATCTGGAGGTGAAAATAGGG	AGGAGTCCCACACAGGGTCT	113,3 %
**MYB**	TCCACACTGCCCAAGTCTCT	AGCAAGCTGTTGTCTTCTTTGA	87,4%
**RPLP0**	CCATCAGCACCACAGCCTTC	GGCGACCTGGAAGTCCAACT	87,9%
**p130**	AGGCCCTGGCTGGAAGTCTGA	GGCCACCTGGGGAAATCTGTTGAA	112,1%
**p107**	TTACCACGAAGCCGGAAGCAGAGG	AGTCATCCCCAATCATCCGAAAAT	102,1%
**RB1**	TATTAGCTAAAGGGGAAGTATT	ACATGGGAGGTGAGAGTTTA	118,4%
***RB1* gene**	GGTTTGGCAGAGAAGGCAGAA	AAAAAGTGGGGAAAGGGTAT	102,3%
murine			
	**forward**	**reverse**	
**MYBL2**	TGAGGCCCGAGAAGCAGAAGAGAA	CAGGGGTTTGGGCATGGTAGAGGA	112,4%
**CCNB1**	GCCAAGCGTGTGCCTGTGA	GCTCTTCGCTGACTTTATTACCA	100,2%
**MKI67**	GCCCAGGCAGTCCATAAAAC	GCGGGGCTGCCAACATCC	92,6%
**muRPL37**	TAGAAGCAAGATGACGAAGG	GTCGACTTCTGAAGGTGGTAG	88,2%
